# Supplementary material for: Adoptability of digital payments for community health workers in peri-urban Uganda: A case study of Wakiso district
Source: PLoS One. 2024 Aug 15;19(8):e0308322. doi: 10.1371/journal.pone.0308322 (PMC11326584; doi:10.1371/journal.pone.0308322)
Supplement: S1 File — (ZIP) [file pone.0308322.s002.zip › S1_File/In-Depth Interview guide.pdf]

IN-DEPTH INTERVIEW GUIDE

Parish:		Date:	
NGO attached to:		Health facility attached to:	
Name of interviewer:		Age:	
Start:		End:	

After a brief introduction to the participant regarding the purpose of the interview, the interviewer will take informed written consent for the interview. Consent will also be requested for audio recording.

Now, I would like to ask you a few questions. Before I start let me stress that there are no right or wrong answers, no desirable or undesirable answers. Please feel free to say what you really think or feel.
I am going to ask you some questions about Digital Health Payments for Community Health Workers. You do not have to answer these questions if you do not want to. As a reminder, your responses will be anonymized, meaning that your name will not be attached to them.

1. Would you like to tell me a bit about yourself, who you are and what you do?
2. Share with me your experience in being paid by IDI or UNEPI using Mobile Money for health activities.
   Probes: *Timeliness, successful of payments, completeness of payments, performance when paid in this manner, enable carrying out other financial tasks like sending money (financial transfer, shopping). Does it vary with the paying organization? How do they compare with cash-based payments?*
3. In your opinion, how easy is it to use Mobile Money or other digital payment systems for receiving health activity payments by UNEPI or IDI? *Probes: How do they compare with cash-based payments? Is it easy to learn how to use the system? Previous experience in using this payment system before apart from health payments? Process of actually getting and using the money.*
4. In your opinion, do you find health payments using Mobile money and other digital payment systems risky? *Probes: Feeling of security of monies while using this payment method, concerns of safety of personal information, concerns of payment shortages.*
5. *How much do you trust Mobile Money or other digital payments for health activities? Trust in payment systems e.g., Mobile network service provider/bank, trust in Mobile Money agents, trust in the payment provider to send payments.*
6. As we conclude, could you share with me anything else about Digital Payments for payment of Community Health Workers that we may not have talked about?
