# Supplementary material for: Adoptability of digital payments for community health workers in peri-urban Uganda: A case study of Wakiso district
Source: PLoS One. 2024 Aug 15;19(8):e0308322. doi: 10.1371/journal.pone.0308322 (PMC11326584; doi:10.1371/journal.pone.0308322)
Supplement: S1 File — (ZIP) [file pone.0308322.s002.zip › S1_File/Key Informant Interview guide.pdf]

KEY INFORMANT INTERVIEW GUIDE

NGO:		Date:	
Name of interviewer:		Age:	
Start:		End:	

After a brief introduction to the participant regarding the purpose of the interview, the interviewer will take informed written consent for the interview. Consent will also be requested for audio recording.

Now, I would like to ask you a few questions. Before I start let me stress that there are no right or wrong answers, no desirable or undesirable answers. Please feel free to say what you really think or feel.
I am going to ask you some questions about Digital Health Payments for Community Health workers. You do not have to answer these questions if you do not want to. As a reminder, your responses will be anonymized, meaning that your name will not be attached to them.

1. Would you like to tell me a bit about yourself, who you are and what you do?
2. Share with me your experience in paying Community Health Workers using Digital methods like Mobile money for payment of Community Health Workers?
   Probes: How do they compare with cash-based payments? Successfulness of payments, Timeliness, completeness of payments.
3. In your opinion, how easy is it to pay Community Health workers using Digital payment (Mobile Money)?
   Probes: How do they compare with cash-based payments? Is it easy to learn how to use the system to process bulk payments for the CHWs? Previous experience in using this payment system for making bulk health payments, Repetition of processed Payments.
4. In your opinion, do you find it risky to use Digital payment systems for payment of Community Health Workers? Probes: Risk of double payments, organizational losses in payment process.
5. As we conclude, could you share with me anything else about Mobile Money or other Digital Payments systems for payment of Community Health Workers that we may not have talked about?
