# Supplementary material for: Adoptability of digital payments for community health workers in peri-urban Uganda: A case study of Wakiso district
Source: PLoS One. 2024 Aug 15;19(8):e0308322. doi: 10.1371/journal.pone.0308322 (PMC11326584; doi:10.1371/journal.pone.0308322)
Supplement: S1 File — (ZIP) [file pone.0308322.s002.zip › S1_File/Questionnaire.pdf]

Socio-demographic data

Name of payment provider

- ○ IDI
- ○ UNEPI

Gender of respondents:

- ○ Female
- ○ Male

Age of respondents in years:

…………………………..

Education:

- ○ No formal education
- ○ Primary
- ○ Secondary
- ○ Bachelor's degree

Previous experience in using digital payment systems:

- ☐ Payment by card
- ☐ Use of Mobile money
- ☐ Online bank transfer
- ☐ Other

How did you learn how to use digital payments systems?

- ☐ Self-taught
- ☐ Help from a family member
- ☐ Employer
- ☐ Colleague

How do you acquire your digital payment from for health activities?

- ☐ Mobile money e.g. AIRTEL, MTN
- ☐ Bank transfers

Scale

I strongly disagree	I disagree	I am not sure	I agree	I strongly agree
1	2	3	4	5

Intention to adopt

1	I used to imagine being paid using digital payment systems	1	2	3	4	5
2	I can use digital payment systems regularly	1	2	3	4	5
3	Using digital payment systems is good for me	1	2	3	4	5
4	Modern life involves the use of digital payment systems	1	2	3	4	5
5	I would recommend digital payment systems to others	1	2	3	4	5
6	I am okay with the costs incurred in digital transactions	1	2	3	4	5
7	I am willing/okay with being paid through digital systems by my employer in the future	1	2	3	4	5

Perceived usefulness

1	With digital payment systems, I always receive my money for the health activities	1	2	3	4	5
2	Digital payments enable me to receive payment on time	1	2	3	4	5
3	Overall, digital payments would support me in coping with work challenges/problems	1	2	3	4	5
5	Digital payments increase my performance at work and I get the expected results	1	2	3	4	5
6	Digital payments enable me to conduct tasks such as money transfers, shopping, more easily	1	2	3	4	5

Perceived ease of use

2	Using digital payments is easier than cash-based payment because it allows me to access my payment faster	1	2	3	4	5
3	I can easily navigate the digital system to access my electronic money	1	2	3	4	5
4	It is easy to learn how to use digital payment systems like Mobile money	1	2	3	4	5
5	Digital payment systems are clear and easily comprehensible to me	1	2	3	4	5

Perceived risk

1	Digital payments are reliable compared to cash-based payments	1	2	3	4	5
2	I am sure of getting my payment when paid using digital systems	1	2	3	4	5
3	I am afraid of making an irrevocable mistake while using digital payment systems	1	2	3	4	5
4	I am concerned about the privacy of my personal information when using my payments systems	1	2	3	4	5
5	I feel that I am in control of my money when paid digitally					

Trust

1	Digital payments service providers like Mobile money operators are credible and safe	1	2	3	4	5
2	Digital payments network providers like MTN, Airtel are widely acknowledged and can be trusted	1	2	3	4	5
3	I trust the digital payment providers (e.g., IDI, UNEPI)	1	2	3	4	5
4	Digital payments systems are reliable in my area of work					
