# Supplementary material for: Adoptability of digital payments for community health workers in peri-urban Uganda: A case study of Wakiso district
Source: PLoS One. 2024 Aug 15;19(8):e0308322. doi: 10.1371/journal.pone.0308322 (PMC11326584; doi:10.1371/journal.pone.0308322)
Supplement: Attachment — Submitted filename: Response to reviewers.docx [file pone.0308322.s003.docx]

July 6^th^, 2024,

Dear Editor,

Thank you for reviewing and considering our manuscript, “Adoptability of digital payments for Community Health Workers in peri-urban Uganda: a case study of Wakiso district” for publication in PLOS ONE. Below, we describe how we have further strengthened our manuscript in response to the journal requirements and reviewers’ comments. We quote reviewer comments in *italics*, and changes to the original manuscript are indicated in text. Pages and line numbers referred to herein are in the revised manuscript labeled, “Manuscript”.

***Journal Requirements:***

*Please ensure that your manuscript meets PLOS ONE's style requirements, including those for file naming.*

1. *Please ensure that your manuscript meets PLOS ONE's style requirements, including those for file naming. The PLOS ONE style templates can be found at*

*https://journals.plos.org/plosone/s/file?id=wjVg/PLOSOne_formatting_sample_main_body.pdf and*

[*https://journals.plos.org/plosone/s/file?id=ba62/PLOSOne_formatting_sample_title_authors_affiliations.pdf*](https://journals.plos.org/plosone/s/file?id=ba62/PLOSOne_formatting_sample_title_authors_affiliations.pdf)

**Response:** The manuscript was formatted according to PLOS ONE’s requirements and file naming also followed the journal requirements

2. *We suggest you thoroughly copyedit your manuscript for language usage, spelling, and grammar. If you do not know anyone who can help you do this, you may wish to consider employing a professional scientific editing service.*

*The American Journal Experts (AJE) (https://www.aje.com/) is one such service that has extensive experience helping authors meet PLOS guidelines and can provide language editing, translation, manuscript formatting, and figure formatting to ensure your manuscript meets our submission guidelines. Please note that having the manuscript copyedited by AJE or any other editing services does not guarantee selection for peer review or acceptance for publication.*

*Upon resubmission, please provide the following:*

*The name of the colleague or the details of the professional service that edited your manuscript*

*A copy of your manuscript showing your changes by either highlighting them or using track changes (uploaded as a *supporting information* file)*

*A clean copy of the edited manuscript (uploaded as the new *manuscript* file)”*

**Response:** The manuscript has been reviewed and edited by a professional English language editor. The copy of the manuscript with track changes has been uploaded as a *supporting information* file.

3. *Thank you for stating the following financial disclosure:*

*"Financial support for this study was obtained through the Digital Health Payments Initiative and Research project (DHPI-R) at Makerere University funded by the Bill&Melinda Gates Foundation (grant number: INV-03047). "*

*Please state what role the funders took in the study. If the funders had no role, please state: ""**The funders had no role in study design, data collection and analysis, decision to publish, or preparation of the manuscript.""*

*If this statement is not correct you must amend it as needed.*

*Please include this amended Role of Funder statement in your cover letter; we will change the online submission form on your behalf.*

**Response: “**The funders had no role in study design, data collection and analysis, decision to publish, or preparation of the manuscript.” The findings and conclusions contained within are those of the authors and do not necessarily reflect the positions or policies of the Bill&Melinda Gates Foundation. The Funder will be given an opportunity to review the acknowledgement statement.

4. *Thank you for stating the following in the Acknowledgments Section of your manuscript:*

*"Financial support for this study was obtained through the Digital Health Payments Initiative and Research project (DHPI-R) at Makerere University funded by the Bill&Melinda Gates Foundation. The findings and conclusions contained within are those of the authors and do not necessarily reflect the positions or policies of the Bill&Melinda Gates Foundation. The funders had no role in the study design, data collection and analysis, or preparation of the manuscript."*

*We note that you have provided funding information that is not currently declared in your Funding Statement. However, funding information should not appear in the Acknowledgments section or other areas of your manuscript. We will only publish funding information present in the Funding Statement section of the online submission form.*

*Please remove any funding-related text from the manuscript and let us know how you would like to update your Funding Statement. Currently, your Funding Statement reads as follows:*

*"Financial support for this study was obtained through the Digital Health Payments Initiative and Research project (DHPI-R) at Makerere University funded by the Bill&Melinda Gates Foundation (grant number: INV-03047)."*

*Please include your amended statements within your cover letter; we will change the online submission form on your behalf.*

**Response:** The statement in acknowledgements section has been revised and now reads, “This Manuscript is based on research funded by the Bill & Melinda Gates Foundation. The findings and conclusions contained within are those of the authors and do not necessarily reflect the positions or policies of the Bill&Melinda Gates Foundation. The Funder will be given an opportunity to review the acknowledgement statement.”

5. *Please include a caption for figure 1.*

***Response:*** *“Figure 1” has been changed to S2_Fig (See line 144 of the revised manuscript) The file had been attached on the first submission.*

6. *Please review your reference list to ensure that it is complete and correct. If you have cited papers that have been retracted, please include the rationale for doing so in the manuscript text, or remove these references and replace them with relevant current references. Any changes to the reference list should be mentioned in the rebuttal letter that accompanies your revised manuscript. If you need to cite a retracted article, indicate the article’s retracted status in the References list and also include a citation and full reference for the retraction notice.*

**Response:** The reference list has once again been updated to ensure completeness.

**Reviewer #1: Review Reports**

1. *The research article is a very good paper which gives good and strong.*

**Response**: Thank you

2. *On the scope” why adoptability? Is that feasible with the country context? Why only CHWs? Why only NGO workers? What about the internet governance rule of Uganda? Why only among those doing campaign? Why peri-urban? Why only polio eradication Programme?*

**Responses**:

*“why adoptability?”:* We chose to assess adoptability because the CHWs have used digital payments in previous community health activities and campaigns as well as in their day to day lives. We, therefore, wanted to see if they are willing to continue using digital payments for future community health projects. This is indicated in the last paragraph of the introduction section, line 105-110.

*“Is that feasible with the country context?”:* Yes, adoptability of digital payments is feasible in Uganda. There are various forms of digital payments in Uganda, however, Mobile Money transfers run by telecommunication companies are the most feasible and readily available. These only requires one to have a phone (smart phone or not) and they can receive and send their money. We have connectivity of telecommunication services including mobile money agents across the country. Banks are also available across the country though not all the CHWs have accounts.

*“Why only CHWs?”* Salaried cadres of the healthcare system in Uganda already receive payments digitally through bank transfers. CHWs, however, who are engaged and paid occasionally, often receive their money as cash. Payment with cash has been associated with several challenges as indicated in the introduction (line 101-104), such as time wasting as the wait for money at facilities since they’re always in large numbers, transportation of cash from the funders to the distribution centers is often a problem and sometimes results in loss of some funds, the accountability is a problem, and the money is usually not received in full amounts. Digital payments will address these problems in this population. In addition, CHWs are the lowest cadre in the health system, with lower literacy levels so, use of banks like for other cadres could be a challenge.

*“Why only NGO workers?”* The study focused on CHWs who had ever been paid digitally and usually, payments are given by NGOs through their various community projects. Therefore, our inclusion criteria included having received payments digitally and the listed NGOs were some of the biggest funders of recent and ongoing community health projects. UNEPI coordinates government immunization programs in which CHWs are often engaged.

*“What about the internet governance rule of Uganda?”* Digital payments systems mostly used in Uganda as well as the mobile money systems being recommended in the current study do not require internet. The issue of vulnerability to internet hacks and scams is therefore not significant. Besides that, CHWs as well as majority of the population in Uganda already use digital payments in their day to day lives. We wanted to see if these same services are adoptable in the health sector for payment of CHWs for their involvement in health activities.

*“Why only among those doing campaign?”* CHWs are basically volunteers. Majority of their paid activities are community health campaigns such as immunization/vaccination. Therefore, at the time of sampling, majority or all of the CHWs who had received digital payments had been engaged in campaign activities (Line 67-73).

*“Why peri-urban?”* Peri-urban because it gives a good representation of both the urban and the not so urban setting as a proxy for rural settings to show the adoptability in both settings. The findings on adoptability in peri-urban areas gives a starting point for the initiation of adoption of digital payments and scale up to other rural areas as guided by phone coverage.

*“Why only polio eradication Programme?”* These were emphasized by WHO when recommending digital payments however these payment modalities can be used in other community health campaigns. Digital payments apply to other health campaigns besides polio eradication programmes.

3. *The abstract lacks clarity*

**Response**: Thank you. The abstract was edited to provide more clarity.

4. *The background can be stronger than this to convince the reader.*

**Response**: Thank you, we have made some additions to the background to make it stronger as guided.

5. *The methods section is relatively good but;
   • Inclusion criteria is weak to cover both study methods*

**Response**: Thank you, this has been addressed. This survey was conducted among CHWs in Wakiso district who were carrying out community health activities in the district supported by various Implementing Organizations while In-depth interviewees were CHWs with varied experiences with digital payments and willing to share and Key informant interviewees were coordinators of CHWs and individuals who played key roles in management of digital payments for CHWs such as payment providers. See lines 135 – 138.

6. *Trustworthiness is not included*

**Response**: Thank you. Trustworthiness is captured under “Trust” which is a main construct in the conceptual model for this study. Trust is explained in the methods, under the variables section as well as in the analysis plan and the results.

7. *Sub themes were not explained*

**Response**: The sub-themes were added to the results section under each of the constructs (themes). See lines 294-368.

8. *Case to variable ratio is great concern= for logistic regression*

**Response**: "The final model had 4 variables and a total of 150 observations which gave an average of 38 cases per variable. This satisfies the working assumption of 10-20 cases per variable in the model. (Ja, 2017)

9. *The reason for selecting TAM should be explained more*

**Response**: The reasons for selecting TAM were explained in the study design between line 145 to 153. We have also added that the TAM could fit the local context.

10. *In the result and consequent sections, the detail character of the respondent is lacking E.g. age*

**Response**: Thank you, details on age and gender have been added in the results section. See Table 3.

11. *The discussion is weak and weakly referenced*

**Response**: Thank you, the comment has been addressed.

12. *Some sections are missing E.g. Conflict of interest*

**Response**: Conflict of interest statement “The authors have declared that no competing interests exist,” was made in the submission portal as guided but the system. The PLOS One formatting guidelines indicate this, “Do not include funding or competing interests information in Acknowledgments” and there seems not to be provision for a section specifically for Conflict of interest in the same guidelines.

13. *Inconsistency and grammar*

**Response**: Thank you, the manuscript has been reviewed by a professional language editor.

14. *IN the background “ to for”*

**Response**: Thank you, this has been edited.

15. *Use of PIN Vs pin*

**Response**: Thank you, this has been edited.


**Reviewer #2: ### Review Comments to the Author**

1. *The study presents valuable insights into the adoptability of digital payments among Community Health Workers (CHWs) in Wakiso district, Uganda. - The methodology appears sound, employing a mixed-methods approach to gather both quantitative and qualitative data, thereby providing a comprehensive understanding of the subject matter. - The findings highlight the significant role of perceived risk and trust in influencing the adoptability of digital payment modalities among CHWs. - The conclusion provides actionable recommendations for stakeholders to enhance the adoption of digital payment methods in the healthcare sector.*

**Response**: Thank you for appreciating

2. *Clarity and Organization: - The abstract succinctly summarizes the study objectives, methods, key findings, and conclusions. - The flow of information is logical and well-organized, guiding the reader through the background, methods, results, and conclusion seamlessly*

**Response**: Thank you for observing this

3. *Methodological Rigor: ** - The use of the Technology Acceptance Model (TAM) framework adds rigor to the study by providing a theoretical basis for assessing the intention to use digital payment modalities. - Factor analysis and logistic regression strengthen the quantitative analysis, allowing for the identification of key determinants influencing adoptability. - Including qualitative data from Digital Payment Coordinators enriches the study by offering insights from stakeholders involved in facilitating digital payments.*

**Response**: Thank you for noticing this

4. *Key Findings: ** - The finding that nearly all CHWs have previous experience with mobile money is noteworthy and underscores the familiarity of digital payment methods among the study population. - The identification of perceived risk and trust as critical factors influencing adoptability aligns with existing literature on technology acceptance and user behavior.
   - The qualitative findings regarding positive experiences with mobile money, despite some delays, provide valuable context for understanding CHWs' attitudes toward digital payments.*

**Response**: Thank you for appreciating

5. ***Recommendations: ** - The recommendations for collaborative efforts between payment providers and service operators to address payment delays and mitigate risks are actionable and align with the identified barriers to adoptability. - Suggesting strategies to enhance trust and alleviate perceived risks, such as transparent communication and risk management protocols, demonstrates a practical approach to promoting digital payment adoption.*

**Response**: Thank you for noticing this

6. ***Overall Impression: ** - The study makes a significant contribution to the literature on digital payment adoption in the healthcare sector, particularly among CHWs in peri-urban settings. - The findings are relevant not only to stakeholders in Wakiso district but also to policymakers and practitioners seeking to leverage digital solutions to improve healthcare delivery and financial inclusion. - Addressing the review comments and incorporating any necessary revisions will enhance the clarity, rigor, and impact of the study.*

**Response**: Thank you for the observations

**References**

Ja, R. (2017). How many independent variables to include BEFORE running logistic regression? Retrieved from <https://www.researchgate.net/post/How-many-independent-variables-to-include-BEFORE-running-logistic-regression/59bbc2ab96b7e4d751391ff0/citation/download>
